# Cross-calibration of two dual-energy X-ray absorptiometry devices for the measurement of body composition in young children

**DOI:** 10.1038/s41598-022-17711-0

**Published:** 2022-08-16

**Authors:** Jaz Lyons-Reid, Timothy Kenealy, Benjamin B. Albert, Kate A. Ward, Nicholas Harvey, Keith M. Godfrey, Shiao-Yng Chan, Wayne S. Cutfield

**Affiliations:** 1 Liggins Institute, The University of Auckland, Private Bag 92019, Auckland, New Zealand; 2 Department of Medicine and Department of General Practice and Primary Health Care, The University of Auckland, Auckland, New Zealand; 3 MRC Lifecourse Epidemiology Centre, University of Southampton, Southampton, UK; 4 NIHR Southampton Biomedical Research Centre, University of Southampton and University Hospital Southampton NHS Foundation Trust, Southampton, UK; 5 Singapore Institute for Clinical Sciences, Agency for Science, Technology and Research (A*STAR), Singapore, Singapore; 6 Human Potential Translational Research Programme, Yong Loo Lin School of Medicine, National University of Singapore, Singapore, Singapore; 7 Department of Obstetrics and Gynaecology, Yong Loo Lin School of Medicine, National University of Singapore, Singapore, Singapore; 8 A Better Start-National Science Challenge, The University of Auckland, Auckland, New Zealand

**Keywords:** Paediatrics, Whole body imaging

## Abstract

This study aimed to cross-calibrate body composition measures from the GE Lunar Prodigy and GE Lunar iDXA in a cohort of young children. 28 children (mean age 3.4 years) were measured on the iDXA followed by the Prodigy. Prodigy scans were subsequently reanalysed using enCORE v17 enhanced analysis (“Prodigy enhanced”). Body composition parameters were compared across three evaluation methods (Prodigy, Prodigy enhanced, iDXA), and adjustment equations were developed. There were differences in the three evaluation methods for all body composition parameters. Body fat percentage (%BF) from the iDXA was approximately 1.5-fold greater than the Prodigy, whereas bone mineral density (BMD) was approximately 20% lower. Reanalysis of Prodigy scans with enhanced software attenuated these differences (%BF: − 5.2% [95% CI − 3.5, − 6.8]; and BMD: 1.0% [95% CI 0.0, 1.9]), although significant differences remained for all parameters except total body less head (TBLH) total mass and TBLH BMD, and some regional estimates. There were large differences between the Prodigy and iDXA, with these differences related both to scan resolution and software. Reanalysis of Prodigy scans with enhanced analysis resulted in body composition values much closer to those obtained on the iDXA, although differences remained. As manufacturers update models and software, researchers and clinicians need to be aware of the impact this may have on the longitudinal assessment of body composition, as results may not be comparable across devices and software versions.

## Introduction

Dual-energy X-ray absorptiometry (DXA) is a tool that allows the estimation of body composition by measuring the attenuation of X-rays^1^. As each tissue type has a characteristic R value, which is the ratio of X-ray attenuation at high and low energy, lean mass (LM), fat mass (FM), and bone mineral content (BMC) can be estimated^1,2^. Bone mineral density (BMD) is subsequently calculated as the BMC for a projected area (i.e., bone area)^3^. In addition to enabling differentiation between fat and fat-free masses, DXA allows regional estimation of body composition. A limitation is that differences exist between device types and software versions, which may be amplified in young children^4–7^. For example, Barbour et al.^4^ demonstrated that among infants measured using a Hologic device, reanalysis using the updated software increased body fat percentage (%BF) by approximately 50%. These differences are of particular importance in longitudinal studies where DXA devices or software may be upgraded over the course of the study or in multi-centre studies where different DXA devices may be available at each site.

GE Lunar is one of two manufacturers of DXA devices, with the iDXA being their most advanced model (introduced in 2005). The iDXA has improved image resolution due to an X-ray source with a higher voltage (100 kV), greater pixel density, and a greater number of detectors^8^. The enhanced algorithms from the iDXA have been modified to enable old scans from the GE Lunar Prodigy to be reanalysed with GE Lunar’s “enhanced” analysis option, introduced with the version 14 release of their enCORE analysis software in 2012. While differences between the iDXA and Prodigy have previously been reported in adults^9–13^, this has not been evaluated in young children. Furthermore, it is unclear how much of the difference observed between old and new scans is related to the software versus differences in scan resolution between the models. Therefore, among a cohort of young children, we aimed first to determine if body composition values are the same when obtained with a GE Lunar Prodigy and with a GE Lunar iDXA; and second, to determine if body composition values from a Prodigy, reanalysed with the enhanced analysis software, are comparable to those obtained using an iDXA.

## Subjects and methods

A sample of children aged 3.4 years (n = 29) was selected from the Auckland site of the Nutritional Intervention Preconception and During Pregnancy to Maintain Healthy Glucose Metabolism and Offspring Health (NiPPeR) study^14^. Children were selected based on good compliance with the DXA protocol (i.e., producing a DXA scan without movement artefact). The NiPPeR trial was registered on 16 July 2015 with ClinicalTrials.gov (NCT02509988, Universal Trial Number U1111-1171-8056); ethics approval was granted by the Northern A Health and Disability Ethics Committee (15/NTA/21/AM20). Written informed consent was obtained from the parents/guardians of the study subjects. All procedures in this study were conducted according to the ethical principles and guidelines laid down in the Declaration of Helsinki.

Children were scanned on a GE Lunar iDXA (enCORE v17, paediatric mode) immediately followed by a scan on a GE Lunar Prodigy (enCORE v17, paediatric mode). It has previously been reported that the effective radiation dose of the iDXA scanner for an infant phantom was 8.9 μSv^15^, and in adults, 4.7 μSv^16^. In comparison, the global average for daily natural background radiation exposure is 6.6 μSv^17^. Therefore, the risk associated with repeat DXA scanning is low.

Before measurement with the DXA machines, standing height was measured three times to the nearest 0.1 cm using a calibrated SECA 213 portable stadiometer (SECA, Hamburg, Germany), and weight was measured once to the nearest 100 g using calibrated SECA 899 scales. Median height, weight, and date of birth were input into the DXA machines prior to measurement.

Both DXA machines were calibrated daily with a manufacturer-specific calibration block phantom and with a spine phantom at regular intervals. Children were measured while lightly clothed, in clothing without metal, lying supine on the measurement bed within the scan limit borders. Feet were rotated inwards slightly, and a Velcro strap was used to hold feet in place. If necessary, the child was swaddled lightly with a thin blanket, ensuring arms and legs remained separated. Each scan was graded according to the degree of movement, with significant movement artefact being excluded from the main analyses (n = 1). Images with minor movement were flagged and sensitivity analyses were run excluding these participants (n = 11). The results of the sensitivity analyses were little changed, so results are reported for the main analyses only.

Three sets of body composition values were obtained: iDXA scan analysed with enCORE v17 and Prodigy scan analysed with enCORE v17 basic and with enCORE v17 enhanced analysis. Total body less head (TBLH)^18^ and regional estimates of body composition are reported for FM, LM, BMC, and bone area, as well as %BF (FM ÷ total mass × 100) and BMD (BMC ÷ bone area).

### Statistical analyses

Subject characteristics and body composition values are reported as means ± SD for continuous variables and n (%) for categorical variables. Differences in body composition values between the three evaluation methods (iDXA, Prodigy basic, and Prodigy enhanced) were assessed using within-subjects ANOVA with Bonferroni post-hoc testing. Differences between the Prodigy and iDXA scans are reported as percentage differences and 95% confidence intervals.

To assess differences between the devices and software versions across a range of body sizes, Bland–Altman analyses were conducted to compare the Prodigy (basic and enhanced) to the iDXA (reference), with results reported as biases (i.e., mean differences) and 95% limits of agreement (LOA). Finally, equations were developed using linear regression to allow measurements made on the Prodigy to be adjusted to be comparable to those made on the iDXA. Prediction equations were developed using leave-one-out cross-validation for FM, LM, BMC, and bone area. Adjusted body composition values were then compared to the reference (iDXA) using paired-samples *t-*tests and Bland–Altman analyses. All tests were two-tailed and were performed within R (R Foundation for Statistical Computing, Vienna, Austria), with *p* values less than 0.05 being considered statistically significant.

## Results

### Population characteristics

29 children were measured on the two DXA devices. Following exclusion of scans with movement artefact (n = 1), the sample comprised of 28 children, described in Table 1. The excluded child was similar in height, weight, BMI, and age (all p > 0.05).

**Table 1:** Characteristics of the included cohort.

All (n = 28)	
Age (years)	3.4 ± 0.2
Height (cm)	98.8 ± 3.9
Height z-score	0.05 ± 1.08
Weight (kg)	15.3 ± 1.9
Weight z-score	0.10 ± 1.01
BMI (kg/m^2^)	15.6 ± 1.2
BMI z-score	0.10 ± 0.92
**Sex**	
Male	11 (39.3%)
Female	17 (60.7%)
**Ethnicity**	
White	15 (53.6%)
Chinese	7 (25.0%)
Indian	4 (14.3%)
Other	2 (7.1%)

Data are mean ± SD for continuous variables and n (%) for categorical variables.

### Comparison of the prodigy and iDXA

The mean body composition values for each measurement condition are summarised in Table 2 and Supplementary Table S1. Within-subjects ANOVA indicated differences between the three scan conditions (*p* < 0.001 for all body composition values). Post-hoc testing revealed differences between the iDXA and Prodigy basic for all body composition parameters. Following reanalysis of Prodigy scans using enhanced analysis, there remained differences between the iDXA and the Prodigy, except for TBLH BMD (− 0.004 g/cm^2^ [95% CI − 0.009, 0.001], p = 0.131), and some regional estimates (Supplementary Table S1).

**Table 2:** Mean ± SD total body less head (TBLH) body composition values from 3.5-year-old children (n = 28) each measured using combinations of two dual-energy X-ray absorptiometry (DXA) devices and two software versions: iDXA scan, Prodigy scan analysed with basic analysis; and Prodigy scan analysed with enhanced analysis.

	iDXA	Prodigy			
		Basic		Enhanced	
	Mean ± SD	Mean ± SD	% difference^a^	Mean ± SD	% difference^a^
TBLH lean mass (g)	8625 ± 1115	9458 ± 1208	9.70 (8.72, 10.67)	8398 ± 1157	− 2.72 (− 3.51, − 1.93)
TBLH fat mass (g)	3731 ± 980	2408 ± 954	− 37.37 (− 41.07, − 33.68)	3897 ± 986	4.77 (3.13, 6.40)
TBLH fat mass (%)	29.2 ± 4.82	19.5 ± 5.57	− 34.49 (− 38.24, − 30.75)	30.7 ± 4.92	5.15 (3.50, 6.80)
TBLH BMC (g)	296 ± 50	237 ± 54	− 20.66 (− 23.07, − 18.24)	314 ± 51	6.36 (5.10, 7.61)
TBLH bone area (cm^2^)	691 ± 68	436 ± 76	− 37.16 (− 39.60, − 34.72)	727 ± 64	5.37 (4.13, 6.61)
TBLH BMD (g/cm^2^)	0.43 ± 0.04	0.54 ± 0.03	26.57 (24.96, 28.19)	0.43 ± 0.04	0.96 (0.02, 1.90)

*%BF* body fat percentage, *BMC* bone mineral content, *BMD* bone mineral density, *FFM* fat-free mass.

^a^Percentage difference (95% CI) in body composition values in reference to the values obtained from the GE Lunar iDXA.

When expressed as percentage differences, Prodigy basic TBLH values were up to 37% different from those obtained on the iDXA (Table 2). Differences were largest for fat mass (kg and %) and bone parameters (BMC, bone area, and BMD), as well as for regional estimates, which were up to 65% different (Table 2 and Supplementary Table S1). When Prodigy scans were reanalysed using enhanced analysis, the percentage differences reduced to < 6.5% for TBLH values and < 15.5% for regional estimates (Table 2 and Supplementary Table S1).

The Bland–Altman analyses are reported in Tables 3 and Table S1 and Supplementary Fig. S1. Compared to the iDXA, Prodigy basic LM was higher by ~ 800 g and FM lower by ~ 1.3 kg, resulting in a difference in total mass of − 550 g and a difference in %BF of − 9.7%. Both bone area and BMC were reduced (− 255 g [95% LOA − 329, − 181] and − 59 [95% LOA − 86, − 32], respectively), although bone area to a greater extent, resulting in greater estimates of BMD (+ 0.11 g/cm^2^ [95% LOA 0.09, 0.13]). A systematic bias for %BF was observed, with differences being greater among those with low %BF. When the Prodigy scans were reanalysed using enhanced analysis, the bias for TBLH LM reduced to less than 250 g, while the bias for FM reduced to almost a tenth of the original value (+ 167 g [95% LOA − 133, 466]). Meanwhile, the bias for BMD was reduced to 0 g/cm^2^. Regional analyses paralleled the TBLH results, with the Prodigy basic having higher LM but lower FM and bone area. Reanalysis of Prodigy files with enhanced analysis attenuated these differences.

**Table 3:** Bland–Altman analysis comparing total body less head (TBLH) body composition parameters of young children measured by dual-energy X-ray absorptiometry (DXA) on the GE Lunar Prodigy, analysed using basic and enhanced analysis in reference to scans obtained on the GE Lunar iDXA.

	Prodigy			
	Basic		Enhanced	
	Bias (95% LOA)	*p*	Bias (95% LOA)	*p*
TBLH lean mass (g)	832 (376, 1288)	0.071	− 227 (− 574, 119)	0.230
TBLH fat mass (g)	− 1323 (− 1699, − 947)	0.478	167 (− 133, 466)	0.841
TBLH fat mass (%)	− 9.7 (− 13.1, − 6.4)	**0.021**	1.5 (− 0.8, − 3.7)	0.660
TBLH BMC (g)	− 59 (− 86, − 32)	0.134	18 (1, 35)	0.709
TBLH bone area (cm^2^)	− 255 (− 329, − 181)	0.295	36 (− 4, 76)	0.313
TBLH BMD (g/cm^2^)	0.11 (0.09, 0.13)	0.070	0.00 (− 0.02, 0.02)	0.374

Significant values are in [bold].

### Adjustment equations

**Figure 1:**
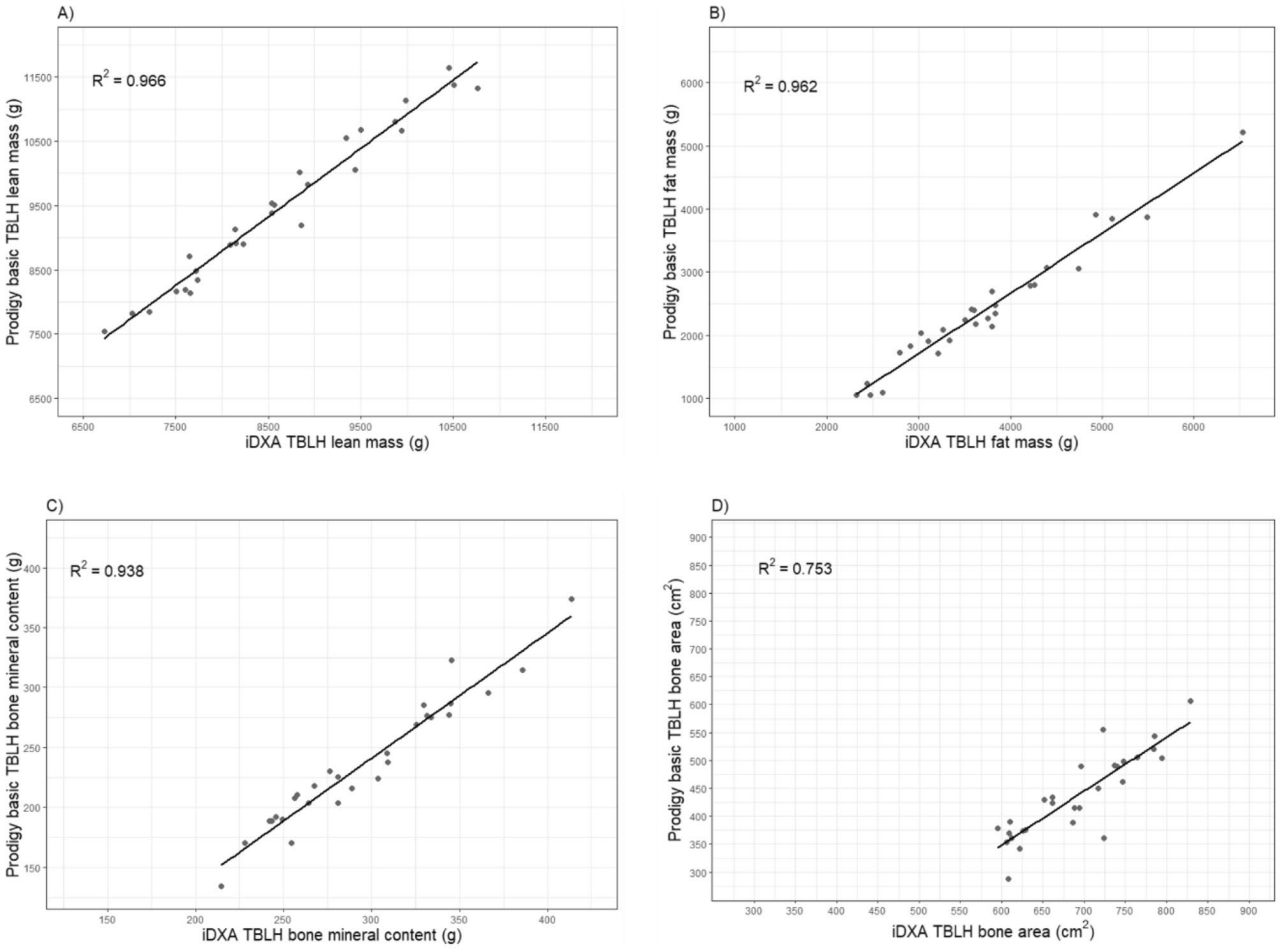
Scatterplots of total body less head (TBLH) estimates of (**A**) lean mass (g), (**B**) fat mass (g), (**C**) bone mineral content (g), and (**D**) bone area (cm^2^) from the GE Lunar Prodigy basic and the GE Lunar iDXA.

Prediction equations were developed to enable adjustment of Prodigy (basic) measurements (Table 4 and Fig. 1). Prediction equations for enhanced measurements are contained within the supplementary file (Supplementary Table 2 and Supplementary Fig. S2).

**Table 4:** Cross-calibration equations between GE Lunar Prodigy basic and GE Lunar iDXA (reference) measurements among 28 young children.

	Slope (95% CI)	Intercept (95% CI)	R^2^
TBLH lean mass (g)	0.91 (0.85, 0.97)	38.53 (− 581.83, 658.89)	0.966
TBLH fat mass (g)	1.01 (0.93, 1.09)	1303.08 (1103.40, 1502.76)	0.962
TBLH bone mineral content (g)	0.90 (0.80, 1.00)	83.41 (61.81, 105.01)	0.938
TBLH bone area (cm^2^)	0.78 (0.60, 0.96)	349.82 (773.60, 926.04)	0.753

When the equations were validated, the adjusted values aligned more closely with iDXA estimates than the reanalysed Prodigy scans (i.e., enhanced Prodigy) did. For example, in comparison to iDXA estimates, Prodigy enhanced LM was 2.7% lower, with a bias of ~ 250 g, whereas adjusted Prodigy LM was almost identical (0.0% [95% CI − 1.8, 1.7]), with a bias of less than 10 g (Table 5).

**Table 5:** Total body less head (TBLH) body composition estimates and results from Bland–Altman analysis comparing body composition parameters from the iDXA and adjusted Prodigy values.

	iDXA	Adjusted prodigy			
	Mean ± SD	Mean ± SD	% difference^a^	Bias (95% LOA)	*p*
TBLH lean mass (g)	8915 ± 1354	8909 ± 1334	0.0 (− 1.8, 1.7)	− 6 (− 444, 432)	0.818
TBLH fat mass (g)	3515 ± 790	3489 ± 887	− 1.1 (− 6.1, 3.9)	− 26 (− 482, 430)	0.265
TBLH BMC (g)	303 ± 59	302 ± 59	− 0.2 (− 3.3, 2.8)	− 1 (− 22, 20)	0.983
TBLH bone area (cm^2^)	709 ± 85	696 ± 69	− 1.4 (− 6.0, 3.1)	− 13 (− 94, 69)	0.297

*BMC* bone mineral content.

^a^Percentage difference (95% CI) in body composition values in reference to the values obtained from the GE Lunar iDXA.

## Discussion

Previous studies have identified differences between DXA models and software versions; however, few have evaluated differences in young children. The International Society for Clinical Densitometry (ISCD) recommend in vitro cross-calibration when comparing devices of the same model but in vivo cross-calibration when comparing devices from different manufacturers^19^. A study comparing two models by the same manufacturer found that spine phantom cross-calibration can be inaccurate compared to in vivo calibration^20^. This is further complicated in body composition studies, as there is a lack of a suitable phantom for cross-calibration of fat and lean masses. Therefore, in our study, we cross-calibrated two GE Lunar DXA systems (Prodigy and iDXA) in vivo among 28 young children and found significant differences between the two devices, even after Prodigy scans were reanalysed with enhanced analysis.

To our knowledge, no previous study has cross-calibrated the Prodigy and iDXA in a cohort of young children (< 5 years). DXA cross-calibration studies in young children are limited; however, a previous study (3–19 years, n = 126) found that FM from the iDXA (v16) was approximately 15% higher in girls and 31% higher in boys in comparison to the GE Lunar DPX-Pro (v9.3). LM was also reduced when measured with the iDXA compared to the DPX-Pro; however, this was only significant in boys^21^. Other studies have compared single Hologic scans reanalysed with updated software and found differences in FM, FFM, and %BF, but no differences in total mass^4,6^. In young children, there are clear differences between device types and software versions; however, the contribution of scan versus software has not previously been evaluated.

We observed differences between the two devices in all parameters, with iDXA %BF being approximately 1.5-fold greater than Prodigy measurements, whereas BMD was ~ 20% lower (Table 2). When we reanalysed the Prodigy scans with enhanced analysis, although differences remained in all estimates except for TBLH BMD, as well as some regional estimates, the percentage differences and biases were substantially reduced (Table 2 and Table S1).

In our study, differences between devices were most substantial among children with low %BF. Shypailo et al.^6^ reanalysed a large number of paediatric scans (n = 1384) obtained with a Hologic QDR-4500 (v11.2) with updated software (v12.1) and observed greater differences in FM and %BF among younger, smaller subjects, and in girls; although, these results may not be relevant to GE Lunar devices given the differences in technology used in the two scanner types^22^. A pilot study in 13 women (20–46 years) found that differences between the iDXA and Prodigy were most substantial among women who were least adipose (< 20 kg FM and < 30% BF)^16^. DXA estimates body composition according to the attenuation of X-ray beams at high and low energy. A limitation of the technology is that DXA can only differentiate between two tissue types simultaneously (i.e., bone vs non-bone, fat vs lean)^1^. In an adult DXA scan, 40 to 45% of pixels will contain bone, fat, and lean tissue, whereas, in children, this percentage is increased^4^. Therefore, improvements to the estimation of body composition in bone-containing tissue will have a greater impact in younger, smaller children. This may also explain why in some cross-validation studies, only regional estimates were affected^9,10^.

Although comparison has not been made between the iDXA and the Prodigy in a cohort of young children, previous studies in adults have found only small differences between the Prodigy and the iDXA, which have not been consistent across body composition parameters and regions, nor in the direction of the difference^9–13^. The variations in software used may partially explain these conflicting results. The studies used Prodigy scanners with enCORE software versions ranging from 6.10 to 16, while the iDXA scanners used enCORE software version 12.3–17^9–13^.

Watson et al.^13,23^ evaluated differences between the iDXA and Prodigy following reanalysis of Prodigy files with enhanced analysis in both adults (20–65 years, n = 69) and school-aged children (6–16 years, n = 124). Among their cohort of children, differences were apparent in all parameters except whole-body, leg, and trunk BMC. Similar to our findings, differences were most pronounced for total FM and LM, which were 0.71 kg (6%) higher and 1.07 kg (3.5%) lower with the Prodigy than the iDXA^23^.

Although they did not compare basic and enhanced analysis in their study of children, among adults, Watson et al.^13^ noted no differences in whole-body FM and LM when Prodigy scans were analysed with basic compared to enhanced analysis. However, the authors observed differences in total BMC and bone area and regional FM and LM (arm FM and leg LM). This contrasts with our study, where substantial differences were noted between Prodigy scans analysed with the two software versions for all parameters. In line with our results, Crabtree et al.^22^ found differences between basic and enhanced analysis when data was pooled from DXA studies involving children aged 4–20 years.

An inherent limitation of using DXA is that although based on basic principles and hence intrinsically accurate, the software used to analyse the data is proprietary. Animal cadaver studies have shown that both the Prodigy and iDXA have good correlation with chemical analysis results, though many body composition parameters were over- or underestimated^24,25^, which may in part be due to differences in animal tissue thickness^26,27^ and FFM hydration^24,26^. The proprietary nature of the software means that we are unable to fully elucidate where differences between the devices may stem from, though our results suggest a larger role of software than instrumentation. However, it is unclear how the enhanced software option that can be applied to Prodigy scans differs from the default iDXA software, and what adjustments are applied to paediatric scans.

An additional limitation of our study is that we could not compare our results to a suitable reference method to determine which of the two DXA scans was most accurate. In early childhood, there is no gold-standard method for assessing body composition. A four-compartment (4C) model may be used as a reference since it provides additional clarification about the composition of the FFM compartment^2^; however, this would have been time- and resource-intensive. Furthermore, air displacement plethysmography body volume measurements (as required for computation of body composition using a 4C model) are currently not optimised for use at this age^28^. Nonetheless, a previous study in adults found that the iDXA aligned more closely with a 4C model than results from the Prodigy, although there was a systematic bias, with FM being overestimated among those with greater FM^13^. This systematic bias in FM was not observed when iDXA measurements were validated against a 4C model in school-aged children, although mean FM was overestimated by 2 kg^23^. The authors also found iDXA to underestimate FFM by 1.3 kg, with this increasing as total FFM increased^23^. Correction of iDXA FFM according to individually measured TBW (i.e., correcting for FFM hydration) resulted in a reduction in limits of agreement and removal of the systematic bias. However, a mean bias of approximately 2 kg remained^23^. In addition to determining which DXA device is more accurate, we acknowledge the need to replicate the adjustment equations in an independent group of children.

In summary, we have conducted the first cross-calibration study of the GE Lunar Prodigy and iDXA in a cohort of young children. There were substantial differences between the iDXA and the Prodigy, which were attenuated following reanalysis of the Prodigy scans with enhanced software. Thus, the same child scanned by the two devices will yield different results in part due to differences in scan resolution but also due to software differences. However, it is difficult to disentangle these differences and to determine which is a more accurate reflection of true body composition. This highlights a key challenge researchers and clinicians face when collecting longitudinal body composition data in children. As manufacturers upgrade devices and software over the duration of a study or clinical observation, it becomes difficult to determine the true trajectory of body composition. Therefore, researchers and clinicians need to consider the manufacturer, model, and software version when conducting DXA scans as results may not be comparable.

## Supplementary Information

Supplementary Information.

## Data Availability

The datasets generated during and/or analysed during the current study are not publicly available as the participants did not consent to open access data sharing and this is an ongoing longitudinal study in which there will be further future analyses conducted but are available from the corresponding author on reasonable request.
