# Supplementary material for: Cross-calibration of two dual-energy X-ray absorptiometry devices for the measurement of body composition in young children
Source: Sci Rep. 2022 Aug 16;12:13862. doi: 10.1038/s41598-022-17711-0 (PMC9381538; doi:10.1038/s41598-022-17711-0)
Supplement: Supplementary file 1 — Supplementary Information. [file 41598_2022_17711_MOESM1_ESM.pdf]

# Cross-calibration of two dual-energy X-ray absorptiometry devices for the measurement of body composition in young children

**Jaz Lyons-Reid[1], Timothy Kenealy[1,2], Benjamin B. Albert[1], Kate A. Ward[3,4], Nicholas Harvey[3,4], Keith M. Godfrey[3,4], Shiao-Yng Chan[5,6,7], Wayne S. Cutfield[1,8*]**

[1]Liggins Institute, The University of Auckland, Auckland, New Zealand

[2]Department of Medicine and Department of General Practice and Primary Health Care, The University of Auckland, Auckland, New Zealand

[3]MRC Lifecourse Epidemiology Centre, University of Southampton, Southampton, United Kingdom

[4]NIHR Southampton Biomedical Research Centre, University of Southampton and University Hospital Southampton NHS Foundation Trust, Southampton, United Kingdom

[5]Singapore Institute for Clinical Sciences, Agency for Science, Technology and Research (A*STAR), Singapore

[6]Human Potential Translational Research Programme, Yong Loo Lin School of Medicine, National University of Singapore, Singapore

[7]Department of Obstetrics & Gynaecology, Yong Loo Lin School of Medicine, National University of Singapore, Singapore

[8]A Better Start – National Science Challenge, The University of Auckland, Auckland, New Zealand

Corresponding author: Wayne S. Cutfield, MD, Liggins Institute, University of Auckland, Private Bag 92019, Auckland, New Zealand. Email: w.cutfield@auckland.ac.nz. Ph: +64 9 923 4476

Running title: Calibration of DXA devices in young children

Competing interests: KMG, SYC, and WSC are part of an academic consortium that has received grants from Société Des Produits Nestlé S.A. outside the submitted work. SYC and KMG have received reimbursement for speaking at conferences sponsored by companies selling nutritional products. All other authors declare no competing interests.

Supplementary Table 1 Regional body composition estimates and results from repeated measures analysis of variance and Bland–Altman analysis comparing body composition parameters of young children (n=28) measured using two dual-energy X-ray absorptiometry (DXA) devices analysed using different software versions: iDXA scan, Prodigy scan analysed with basic analysis; and Prodigy scan analysed with enhanced analysis.

| | $p^{\$}$ | iDXA | Prodigy | | | | | | | | |
|---|---|---|---|---|---|---|---|---|---|---|---|
| | | | Basic | | | | Enhanced | | | | |
| | | Mean ± SD | Mean ± SD | $p^{\&}$ | % difference[@] | Bias (95% LOA)[+] | Mean ± SD | $p^{\wedge}$ | $p^{\#}$ | % difference | Bias (95% LOA) |
| Lean mass (g) | | | | | | | | | | | |
| - Arms | **<0.001** | 862 ± 127 | 1009 ± 177 | **<0.001** | 17.2 (12.3, 22.0) | 147 (-69, 363) | 835 ± 149 | **<0.001** | 0.245 | -3.2 (-6.6, 0.2) | -27 (-181, 127) |
| - Left arm | **<0.001** | 427 ± 67 | 505 ± 89 | **<0.001** | 18.6 (13.6, 23.6) | 78 (-29, 186)[*] | 416 ± 82 | **<0.001** | 0.786 | -2.5 (-7.0, 1.9) | -11 (-108, 86) |
| - Right arm | **<0.001** | 436 ± 65 | 504 ± 91 | **<0.001** | 16.0 (10.5, 21.6) | 69 (-52, 190)[*] | 419 ± 75 | **<0.001** | 0.143 | -3.7 (-7.3, -0.1) | -16 (-97, 64) |
| - Legs | **<0.001** | 2815 ± 425 | 3156 ± 456 | **<0.001** | 12.3 (10.4, 14.3) | 341 (82, 600) | 2534 ± 468 | **<0.001** | **<0.001** | -10.4 (-12.3, -8.5) | -282 (-503, -60)[*] |
| - Left leg | **<0.001** | 1398 ± 216 | 1 566 ± 225 | **<0.001** | 12.2 (10.3, 14.2) | 167 (38, 296) | 1266 ± 244 | **<0.001** | **<0.001** | -9.9 (-12.0, -7.7) | -132 (-254, -10)[*] |
| - Right leg | **<0.001** | 1417 ± 211 | 1591 ± 233 | **<0.001** | 12.4 (10.2, 14.6) | 174 (26, 321) | 1268 ± 229 | **<0.001** | **<0.001** | -10.9 (-12.8, -9.0) | -150 (-270, -29) |
| - Trunk | **<0.001** | 4948 ± 616 | 5292 ± 630 | **<0.001** | 7.1 (5.8, 8.3) | 344 (38, 650) | 5029 ± 599 | **<0.001** | **0.036** | 1.8 (0.5, 3.0) | 81 (-232, 394) |
| Fat mass (g) | | | | | | | | | | | |
| - Arms | **<0.001** | 636 ± 151 | 240 ± 137 | **<0.001** | -64.4 (-59.0, -59.7) | -396 (-521, -271) | 707 ± 177 | **<0.001** | **<0.001** | 11.4 (8.0, 14.8) | 71 (-46, 188)[*] |
| - Left arm | **<0.001** | 311 ± 78 | 119 ± 67 | **<0.001** | -63.7 (-68.1, -59.3) | -192 (-256, -128) | 352 ± 86 | **<0.001** | **<0.001** | 13.6 (9.9, 17.3) | 40 (-17, 97) |
| - Right arm | **<0.001** | 325 ± 75 | 121 ± 71 | **<0.001** | -65.0 (-69.9, -60.0) | -204 (-272, -136) | 355 ± 92 | **<0.001** | **<0.001** | 9.4 (5.6, 13.2) | 31 (-40, 101)[*] |
| - Legs | **<0.001** | 1705 ± 433 | 1158 ± 403 | **<0.001** | -33.5 (-36.6, -30.5) | -547 (-717, -377) | 1774 ± 386 | **<0.001** | **0.003** | 5.1 (2.6, 7.6) | 69 (-122, 259)[*] |
| - Left leg | **<0.001** | 849 ± 217 | 575 ± 202 | **<0.001** | -33.6 (-36.8, -30.4) | -273 (-371, -176) | 873 ± 192 | **<0.001** | 0.105 | 4.0 (1.1, 7.0) | 24 (-90, 138)[*] |
| - Right leg | **<0.001** | 856 ± 219 | 582 ± 201 | **<0.001** | -33.4 (-36.5, -30.3) | -274 (-368, -181)[*] | 901 ± 197 | **<0.001** | **<0.001** | 6.2 (3.5, 8.9) | 44 (-62, 150)[*] |
| - Trunk | **<0.001** | 1390 ± 433 | 1010 ± 444 | **<0.001** | -29.6 (-34.5, -24.6) | -379 (-630, -128) | 1417 ± 450 | **<0.001** | 0.801 | 1.9 (-1.6, 5.3) | 27 (-222, 277) |
| Fat mass (%) | | | | | | | | | | | |
| - Arms | **<0.001** | 40.9 ± 6.1 | 18.1 ± 7.2 | **<0.001** | -57.1 (-61.9, -52.2) | -22.9 (-30.6, -15.2) | 44.1 ± 6.2 | **<0.001** | **<0.001** | 8.2 (4.8, 11.6) | 3.2 (-3.3, 9.7) |
| - Left arm | **<0.001** | 40.6 ± 6.4 | 18.1 ± 7.2 | **<0.001** | -56.8 (-61.5, -52.0) | -22.6 (-30.3, -14.9) | 44.2 ± 6.8 | **<0.001** | **<0.001** | 9.2 (5.1, 13.2) | 3.5 (-4.3, 11.3) |
| - Right arm | **<0.001** | 41.2 ± 5.6 | 18.1 ± 7.2 | **<0.001** | -57.3 (-62.3, -52.3) | -23.2 (-31.5, -14.8) | 44.1 ± 6.1 | **<0.001** | **<0.001** | 7.4 (4.0, 10.9) | 2.9 (-3.9, 9.7) |
| - Legs | **<0.001** | 36.5 ± 4.9 | 25.8 ± 5.8 | **<0.001** | -30.1 (-33.3, -26.8) | -10.7 (-15.0, -6.4)[*] | 40.0 ± 5.1 | **<0.001** | **<0.001** | 9.9 (7.4, 12.5) | 3.5 (-0.9, 8.0) |
| - Left leg | **<0.001** | 36.5 ± 4.9 | 25.8 ± 5.8 | **<0.001** | -30.1 (-33.4, -26.8) | -10.7 (-15.3, -6.1)[*] | 39.7 ± 5.5 | **<0.001** | **<0.001** | 8.9 (6.0, 11.8) | 3.2 (-2.1, 8.4) |
| - Right leg | **<0.001** | 36.4 ± 5.0 | 25.7 ± 5.8 | **<0.001** | -30.0 (-33.3, -26.7) | -10.7 (-15.0, -6.3) | 40.3 ± 4.9 | **<0.001** | **<0.001** | 11.0 (8.4, 13.6) | 3.9 (-0.6, 8.3) |
| - Trunk | **<0.001** | 21.3 ± 4.8 | 15.5 ± 5.4 | **<0.001** | -28.9 (-33.8, -24.0) | -5.8 (-9.6, 2.0) | 21.3 ± 5.0 | **<0.001** | 1.000 | 0.1 (-3.2, 3.3) | 0.0 (-3.5, 3.5) |
| BMC (g) | | | | | | | | | | | |
| - Arms | **<0.001** | 46 ± 8 | 32 ± 9 | **<0.001** | -32.8 (-36.3, -29.3) | -15 (-21, -9) | 53 ± 10 | **<0.001** | **<0.001** | 14.1 (10.9, 17.3) | 6 (-1, 14) |

| | $p^{\$}$ | iDXA | Prodigy | | | | | | | | |
|---|---|---|---|---|---|---|---|---|---|---|---|
| | | | Basic | | | | Enhanced | | | | |
| | | Mean ± SD | Mean ± SD | $p^{\&}$ | % difference$^{@}$ | Bias (95% LOA)$^{+}$ | Mean ± SD | $p^{\wedge}$ | $p^{\#}$ | % difference | Bias (95% LOA) |
| - Left arm | **<0.001** | 23 ± 5 | 16 ± 4 | **<0.001** | -31.1 (-34.4, -27.7) | -7 (-10, -4) | 27 ± 5 | **<0.001** | **<0.001** | 15.5 (12.0, 18.9) | 3 (0, 7) |
| - Right arm | **<0.001** | 23 ± 4 | 16 ± 5 | **<0.001** | -34.4 (-38.6, -30.3) | -8 (-12, -4)$^{*}$ | 26 ± 5 | **<0.001** | **<0.001** | 12.9 (9.1, 16.7) | 3 (-2, 8)$^{*}$ |
| - Legs | **<0.001** | 115 ± 23 | 97 ± 24 | **<0.001** | -16.0 (-18.6, -13.5) | -18 (-30, -5) | 123 ± 20 | **<0.001** | **<0.001** | 8.4 (5.4, 11.5) | 8 (-3, 20)$^{*}$ |
| - Left leg | **<0.001** | 57 ± 12 | 48 ± 12 | **<0.001** | -16.0 (-18.8, -13.2) | -9 (-16, -2) | 61 ± 10 | **<0.001** | **<0.001** | 7.4 (3.9, 10.8) | 3 (-3, 10)$^{*}$ |
| - Right leg | **<0.001** | 58 ± 11 | 49 ± 12 | **<0.001** | -16.0 (-18.6, -13.5) | -9 (-15, -3) | 63 ± 11 | **<0.001** | **<0.001** | 9.5 (6.5, 12.5) | 5 (-1, 11) |
| - Trunk | **<0.001** | 135 ± 21 | 108 ± 23 | **<0.001** | -20.4 (-23.2, -17.7) | -27 (-44, -10) | 138 ± 24 | **<0.001** | 0.105 | 2.4 (0.1, 4.7) | 3 (-12, 19) |
| Bone area ($cm^2$) | | | | | | | | | | | |
| - Arms | **<0.001** | 135 ± 18 | 67 ± 16 | **<0.001** | -50.3 (-53.4, -47.3) | -67 (-91, -44) | 147 ± 16 | **<0.001** | **<0.001** | 9.8 (6.5, 13.1) | 12 (-9, 33) |
| - Left arm | **<0.001** | 66 ± 11 | 34 ± 8 | **<0.001** | -48.5 (-51.4, -45.6) | -32 (-45, -19)$^{*}$ | 73 ± 9 | **<0.001** | **<0.001** | 11.7 (7.7, 15.8) | 7 (-5, 19) |
| - Right arm | **<0.001** | 69 ± 8 | 33 ± 8 | **<0.001** | -52.0 (-55.6, -48.4) | -35 (-49, -22) | 74 ± 9 | **<0.001** | **<0.001** | 8.2 (4.5, 11.9) | 5 (-7, 18) |
| - Legs | **<0.001** | 255 ± 31 | 169 ± 32 | **<0.001** | -34.1 (-36.8, -31.4) | -86 (-118, -54) | 267 ± 24 | **<0.001** | **<0.001** | 5.1 (2.4, 7.8) | 12 (-17, 40)$^{*}$ |
| - Left leg | **<0.001** | 128 ± 16 | 84 ± 16 | **<0.001** | -34.2 (-37.1, -31.2) | -43 (-62, -25) | 131 ± 12 | **<0.001** | 0.130 | 3.5 (0.5, 6.6) | 4 (-14, 21)$^{*}$ |
| - Right leg | **<0.001** | 127 ± 15 | 85 ± 16 | **<0.001** | -33.9 (-36.7, -31.2) | -43 (-59, -27) | 135 ± 13 | **<0.001** | **<0.001** | 6.8 (3.7, 9.9) | 8 (-8, 24) |
| - Trunk | **<0.001** | 301 ± 26 | 200 ± 32 | **<0.001** | -33.8 (-36.5, -31.0) | -101 (-143, -60) | 313 ± 30 | **<0.001** | **<0.001** | 4.0 (2.7, 5.3) | 12 (-9, 33) |
| BMD ($g/cm^2$) | | | | | | | | | | | |
| - Arms | **<0.001** | 0.34 ± 0.04 | 0.47 ± 0.03 | **<0.001** | 36.1 (32.2, 40.1) | 0.12 (0.07, 0.18) | 0.36 ± 0.04 | **<0.001** | **0.015** | 4.2 (1.5, 6.8) | 0.01 (-0.03, 0.06) |
| - Left arm | **<0.001** | 0.35 ± 0.04 | 0.47 ± 0.03 | **<0.001** | 34.5 (30.4, 38.6) | 0.12 (0.06, 0.18) | 0.36 ± 0.04 | **<0.001** | 0.136 | 3.8 (0.4, 7.2) | 0.01 (-0.05, 0.07) |
| - Right arm | **<0.001** | 0.34 ± 0.04 | 0.46 ± 0.04 | **<0.001** | 37.6 (33.4, 41.9) | 0.13 (0.06, 0.19) | 0.35 ± 0.04 | **<0.001** | **0.003** | 4.5 (2.2, 6.8) | 0.02 (-0.03, 0.05) |
| - Legs | **<0.001** | 0.45 ± 0.05 | 0.57 ± 0.04 | **<0.001** | 27.8 (25.9, 29.8) | 0.12 (0.09, 0.15) | 0.46 ± 0.05 | **<0.001** | **<0.001** | 3.2 (2.0, 4.3) | 0.01 (-0.01, 0.04) |
| - Left leg | **<0.001** | 0.44 ± 0.05 | 0.57 ± 0.04 | **<0.001** | 28.1 (25.9, 30.4) | 0.12 (0.09, 0.16) | 0.46 ± 0.05 | **<0.001** | **<0.001** | 3.8 (2.3, 5.2) | 0.02 (-0.02, 0.05) |
| - Right leg | **<0.001** | 0.45 ± 0.05 | 0.57 ± 0.05 | **<0.001** | 27.6 (25.3, 29.8) | 0.12 (0.09, 0.16) | 0.46 ± 0.05 | **<0.001** | **0.008** | 2.7 (1.1, 4.2) | 0.01 (-0.02, 0.05) |
| - Trunk | **<0.001** | 0.45 ± 0.04 | 0.54 ± 0.03 | **<0.001** | 20.4 (18.6, 22.2) | 0.09 (0.06, 0.12) | 0.44 ± 0.04 | **<0.001** | **0.047** | -1.6 (-2.9, -0.3) | -0.01 (-0.04, 0.02) |

Abbreviations: BMC, bone mineral content; BMD, bone mineral density; FFM, fat-free mass; LOA, limits of agreement. $^{@}$Percentage difference (95% CI) in body composition values in reference to the values obtained from the GE Lunar iDXA. $^{\$}$*p* value for repeated measures analysis of variance comparing the three measurement conditions: GE Lunar iDXA, GE Lunar Prodigy (basic), and GE Lunar Prodigy (enhanced). $^{\&}$Bonferroni adjusted p value for the comparison of the GE Lunar iDXA and the GE Lunar Prodigy (basic). $^{\wedge}$Bonferroni adjusted p value for the comparison of the GE Lunar Prodigy (basic) and the GE Lunar Prodigy (enhanced). $^{\#}$Bonferroni adjusted p value for the comparison of the GE Lunar iDXA and the GE Lunar Prodigy (enhanced). $^{+}$Bias and 95% LOA from Bland–Altman analysis of body composition values in reference to the values obtained from the GE Lunar iDXA. $^{*}$Statistically significant regression line from Bland–Altman analysis of body composition values in reference to the values obtained from the GE Lunar iDXA.

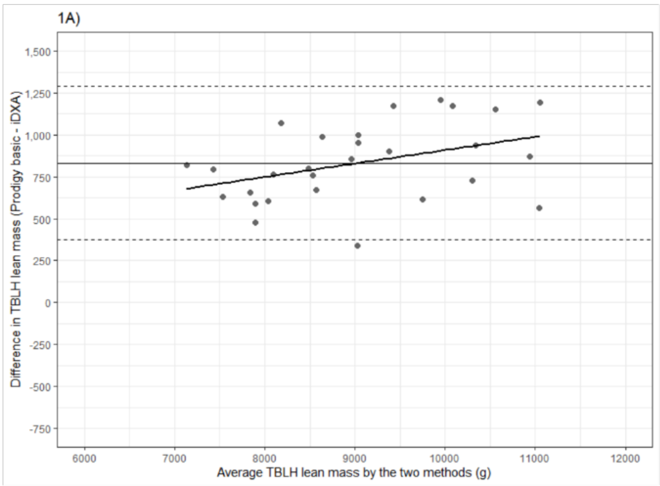
1A)
Difference in TBLH lean mass (Prodigy basic - iDXA)
Average TBLH lean mass by the two methods (g)

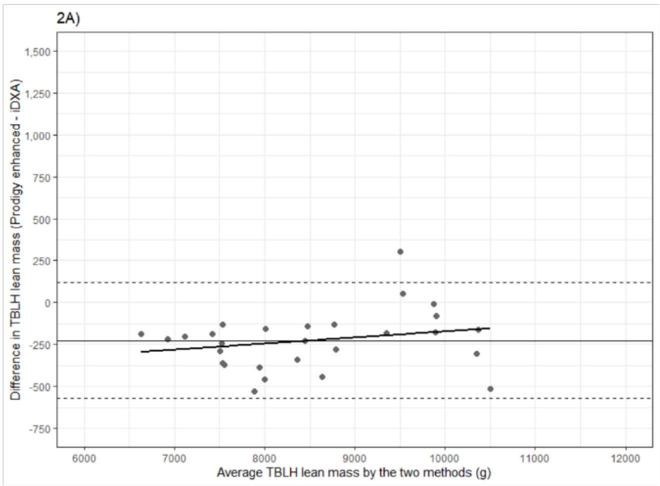
2A)
Difference in TBLH lean mass (Prodigy enhanced - iDXA)
Average TBLH lean mass by the two methods (g)

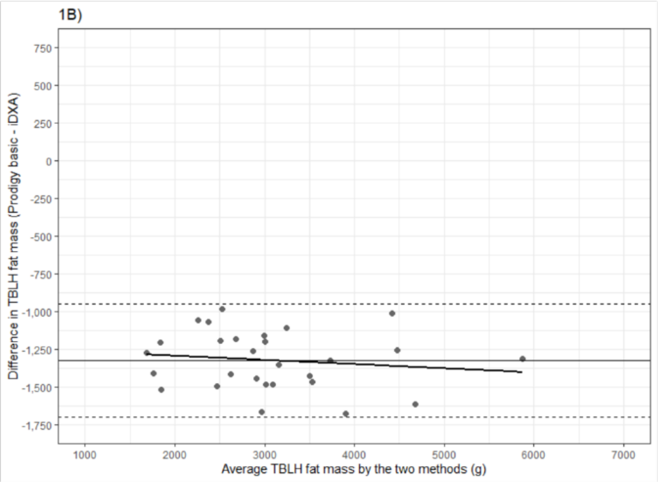
1B)
Difference in TBLH fat mass (Prodigy basic - iDXA)
Average TBLH fat mass by the two methods (g)

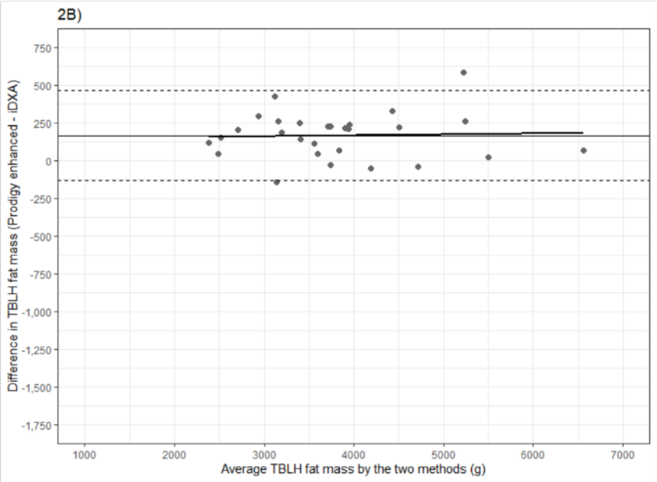
2B)
Difference in TBLH fat mass (Prodigy enhanced - iDXA)
Average TBLH fat mass by the two methods (g)

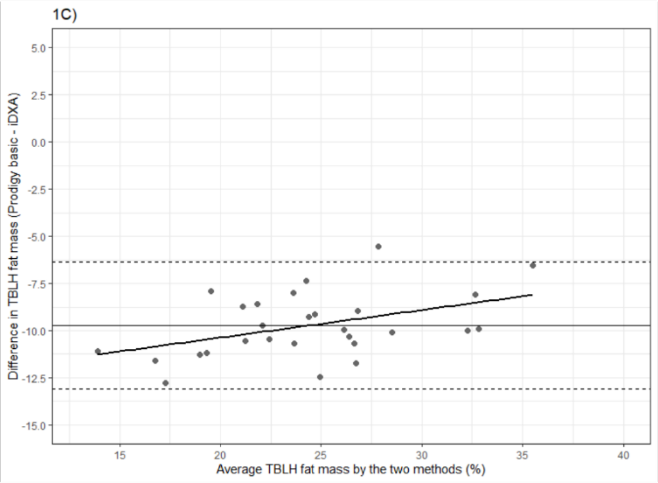
1C)
Difference in TBLH fat mass (Prodigy basic - iDXA)
Average TBLH fat mass by the two methods (%)

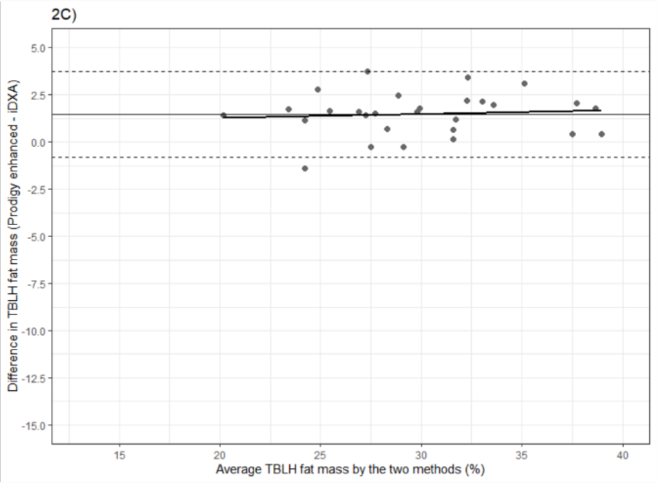
2C)
Difference in TBLH fat mass (Prodigy enhanced - iDXA)
Average TBLH fat mass by the two methods (%)

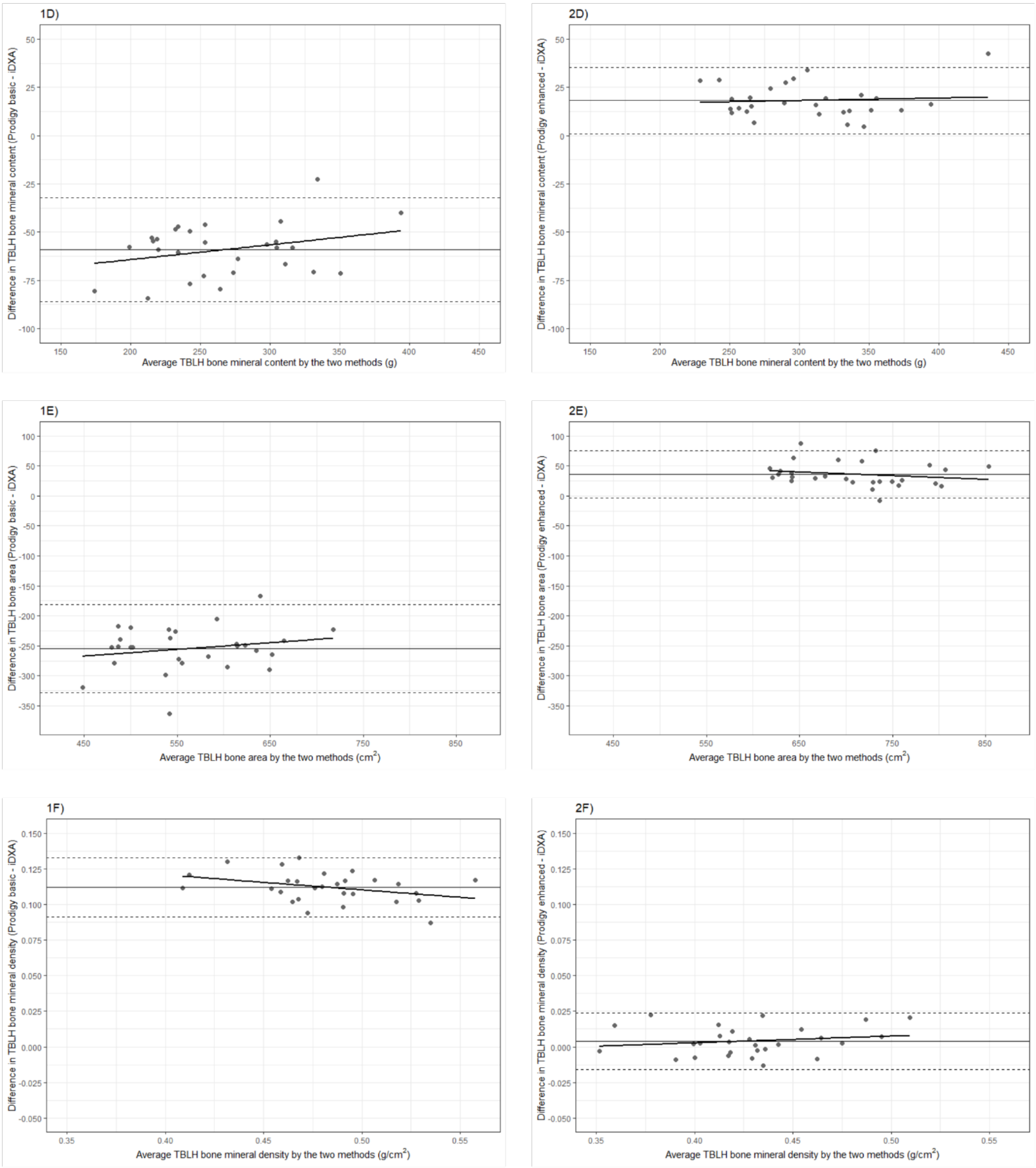


Supplementary Figure 1 Bland–Altman plots comparing total body less head (TBLH) body composition parameters of young children (n=28) measured on the GE Lunar iDXA (reference) and 1) the GE Lunar Prodigy (basic) and 2) the GE Lunar Prodigy (enhanced). Body composition parameters are A) lean mass, B) fat mass, C) fat mass percentage, D) bone mineral content, E) bone area, and F) bone mineral density.

Supplementary Table 2 Cross-calibration equations between GE Lunar Prodigy enhanced and GE Lunar iDXA (reference) measurements among 28 young children.

| | Slope (95% CI) | Intercept (95% CI) | $R^2$ |
|---|---|---|---|
| TBLH lean mass (g) | 0.95 (0.89, 1.01) | 619.60 (145.52, 1093.69) | 0.977 |
| TBLH fat mass (g) | 0.98 (0.92, 1.04) | -96.18 (-333.77, 141.41) | 0.976 |
| TBLH bone mineral content (g) | 0.97 (0.91, 1.03) | -9.52 (-30.41, 11.37) | 0.970 |
| TBLH bone area ($cm^2$) | 1.01 (0.89, 1.13) | -46.22 (-134.44, 42.00) | 0.912 |

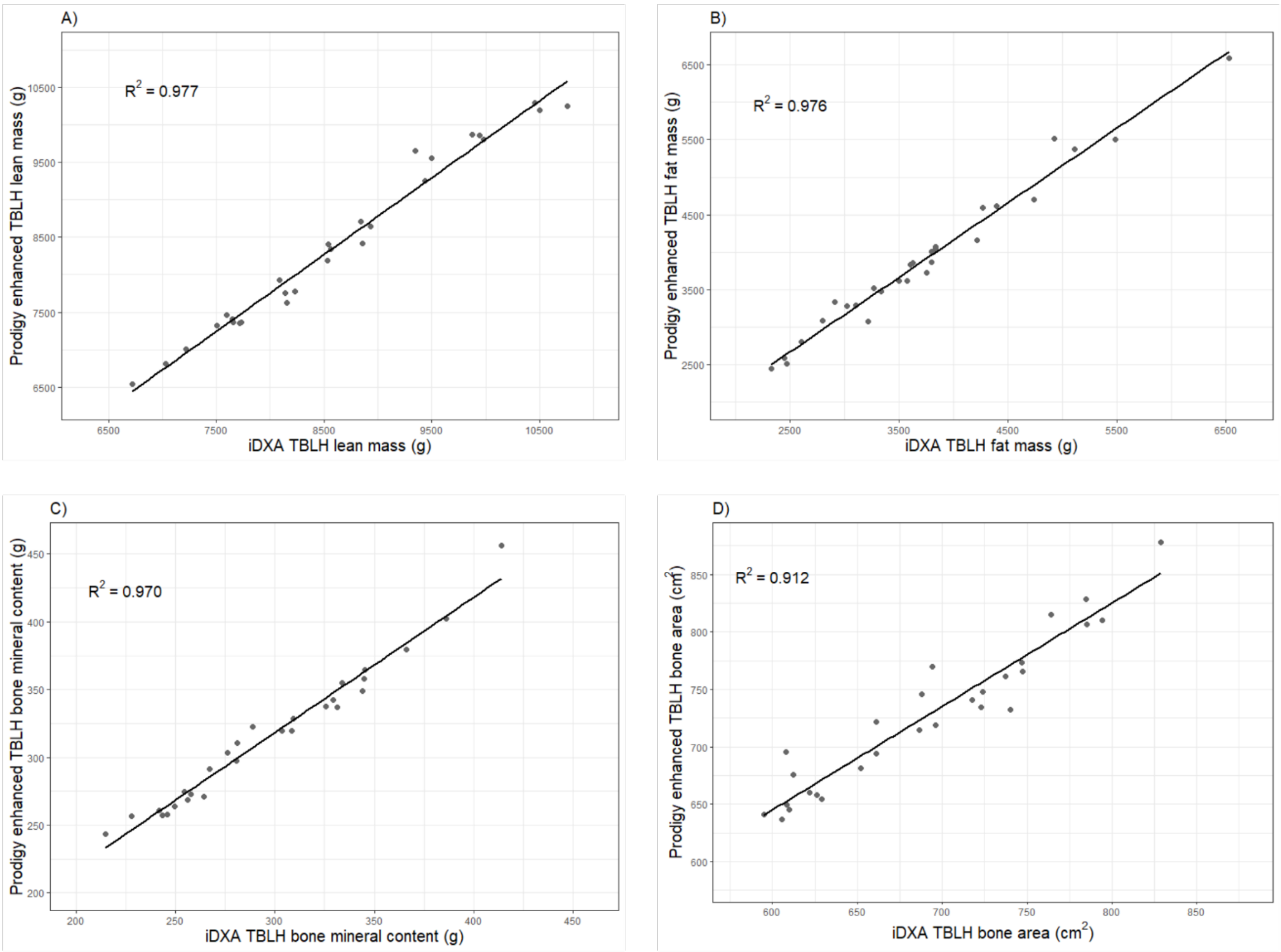


Supplementary Figure 2 Scatterplots of total body less head (TBLH) estimates of A) lean mass (g), B) fat mass (g), C) bone mineral content (g), and D) bone area ($cm^2$) from the GE Lunar Prodigy enhanced and the GE Lunar iDXA.
